# Molecular Dynamics Simulation reveals the mechanism by which the Influenza Cap-dependent Endonuclease acquires resistance against Baloxavir marboxil

**DOI:** 10.1038/s41598-019-53945-1

**Published:** 2019-11-25

**Authors:** Ryunosuke Yoshino, Nobuaki Yasuo, Masakazu Sekijima

**Affiliations:** 1 Transborder Medical Research Center, University of Tsukuba, 1-1-1 Tennodai, Tsukuba, Ibaraki, 305-8577 Japan; 2 Center for Computational Sciences, University of Tsukuba, 1-1-1 Tennodai, Tsukuba, Ibaraki, 305-8577 Japan; 3 Advanced Computational Drug Discovery Unit, Tokyo Institute of Technology, 4259-J3-23 Nagatsutacho, Midori-ku, Yokohama, Kanagawa, 226-8501 Japan

**Keywords:** Protein analysis, Protein function predictions

## Abstract

Baloxavir marboxil (BXM), an antiviral drug for influenza virus, inhibits RNA replication by binding to RNA replication cap-dependent endonuclease (CEN) of influenza A and B viruses. Although this drug was only approved by the FDA in October 2018, drug resistant viruses have already been detected from clinical trials owing to an I38 mutation of CEN. To investigate the reduction of drug sensitivity by the I38 mutant variants, we performed a molecular dynamics (MD) simulation on the CEN-BXM complex structure to analyze variations in the mode of interaction. Our simulation results suggest that the side chain methyl group of I38 in CEN engages in a CH-pi interaction with the aromatic ring of BXM. This interaction is abolished in various I38 mutant variants. Moreover, MD simulation on various mutation models and binding free energy prediction by MM/GBSA method suggest that the I38 mutation precludes any interaction with the aromatic ring of BXA and thereby reduces BXA sensitivity.

## Introduction

Influenza, a severe acute respiratory infection is estimated to affect 5 to 10% of adults and 20 to 30% of children^1^. Moreover, it causes three to five million severe cases and approximately one million deaths worldwide annually^1^. Influenza virus undergoes “antigenic drift,” wherein minor changes in the antigen structure result in the emergence of new viruses, which are able to evade immune recognition and can cause serious pandemics^2^. To treat influenza, development and improvement of antiviral drugs and vaccines against the emerging viruses are required^3^.

Baloxavir marboxil (BXM), with the trade name Xofluza, has been developed by Shionogi Inc and approved by the FDA in October 2018^4,5^. BXM is a prodrug that is converted to the active form, baloxavir acid (BXA) by the enzyme arylacetamide deacetylase^6^. BXA acts by inhibiting the Cap-dependent Endonuclease (CEN) of influenza A and B viruses^5^. CEN is a part of a PA subunit, which constitutes the RNA-dependent RNA polymerase (RdRp) of influenza virus^7–10^. BXA inhibits mRNA synthesis by binding to RdRp and prevents the replication of the influenza virus.

However, influenza viruses resistant to BXA have been detected in samples obtained from phase II and III BXM clinical trials, and it was revealed that the isoleucine-38 (I38) of CEN from BXA resistant viruses is mutated^5^. These I38 mutations in the CEN protein of BXA resistant viruses^5,11^. Moreover, patients infected with viruses containing these mutations have been reported to take more time to recover from the symptoms of influenza include I38T, I38F and I38M^5,11^. These mutations have been shown to exhibit reduced BXA sensitivity of CEN by 10- to 50-fold (EC_50_)^5^. Therefore, in order to improve the efficacy of inhibitors against the mutated viruses, it is necessary to investigate the molecular mechanism of CEN desensitization.

In drug discovery and lead optimization, computational method is a highly effective technique and has been widely applied^12–17^. Typically, computational methods are classified into two types: ligand-based (LB) and structure-based (SB) methods. LB method is based on physical properties and structural information obtained from known active and inactive compounds^18,19^. Quantitative structure activity relationship (QSAR), similarity search and machine learning are representative methods of LB. In contrast, SB method is used when structural information of the target molecule is available^20^. Molecular docking is most often used as a SB method for discovering new compounds^21,22^. This method can predict the mode of binding of a compound to the target molecule by score function, and can narrow down drug candidates from a large number of compounds.

Molecular dynamics (MD) simulation is also used in drug discovery and optimization^23–27^. This method considers the flexibility of the macromolecule based on Newtonian principles and can be applied to various biomolecules, such as protein, nucleic acid, and membranes^28–31^. In drug optimization, MD simulation is generally used to analyze the protein-ligand interaction or the binding free energy. Protein-ligand interaction information is analyzed based on protein-ligand complex ensembles collected during MD simulation. The interaction information obtained by MD simulation can be utilized for compound optimization^32^. The molecular mechanics and generalized born surface area (MM/GBSA) method is typically used to predict the binding free energy of a compound binding to a biological macromolecule^33–35^. MM/GBSA based on MD simulation results, is typically used to search for a new ligand or to optimize a ligand^33^. Moreover, this method can analyze the variation in the sensitivity of a ligand caused by mutations in amino acid residues of the protein^36^.

In the present study, we performed MD simulations to analyze the protein-ligand interaction between the CEN of influenza virus A (pH1N1) with BXA. We further evaluated the binding free energy using the MM/GBSA method. Moreover, we prepared 19 different CEN-BXA models with 19 different I38 mutations modeled in the structure and performed MD simulation and MM/GBSA on these models. We compared the interaction patterns and the binding free energies of all these mutants with those of the wild-type CEN-BXA complex.

## Results

### MD simulation for interaction analysis

**Figure 1:**
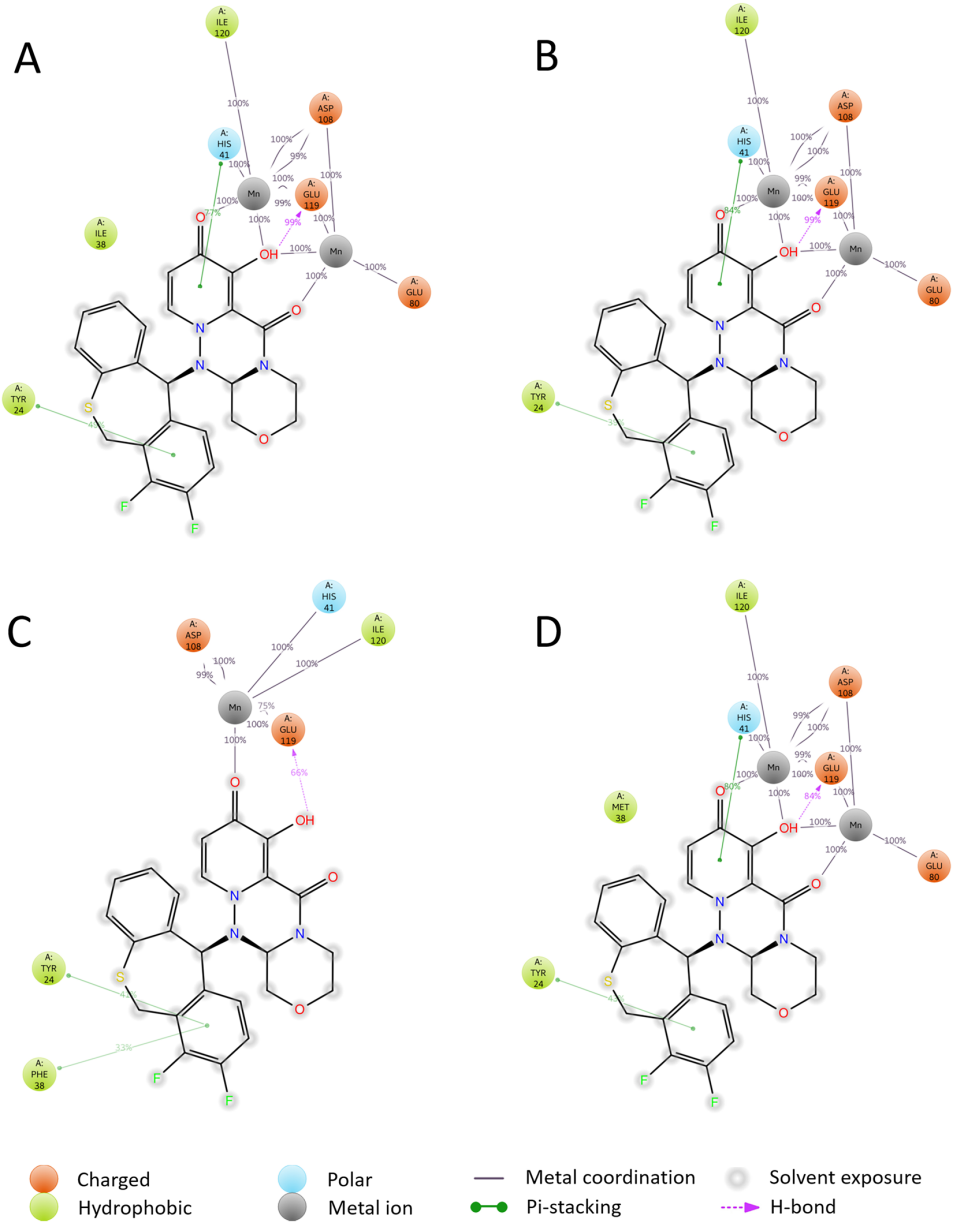
2D summary of interaction analysis results of wild-type and mutant CEN-BXA complexes. The interaction pairs that occur during more than 30% of the simulation time are included. (**A**) Interaction of BXA with CEN (PDB entry: 6FS6), (**B**) Interaction between BXA and CEN with I38T mutation (PDB entry: 6FS7), C: Interaction between BXA and CEN with I38F mutation, D: Interaction between BXA and CEN with I38M mutation.

To analyze interactions between CEN and BXA, we conducted MD simulations on the CEN-BXA complex model. Detailed information regarding the MD simulation is presented in Supplementary Fig. 1. Figure 1A shows a 2D summary of the interaction analysis results of CEN-BXA (PDBID: 6FS6, I38). These interactions occurred with a probability of over 30% during simulation. MD simulations of CEN-BXA complex suggest that Tyr24 and His41 interact with BXA by pi-stacking and Glu119 forms hydrogen bonds with BXA. Moreover, Ile38 is placed adjacent to the aromatic ring of BXA in the simulation. On the contrary, BXA interacts with Glu80, Asp108, and Ile120 via two Mn^2+^ ions. Glu80 and Ile120 have 1 route to interact with the carbonyl or hydroxyl group of BXA; Asp108 and Glu119 have 3 routes to interact with BXA via each Mn^2+^ ion. His41 also has 1 route to interact with the carbonyl or hydroxyl group of BXA via Mn^2+^ ions. Additionally, these ionic interactions via Mn^2+^ ions maintain an appearance probability of over 99%. Therefore, the MD simulation results suggest that the carbonyl and hydroxyl groups of BXA are necessary for interacting with CEN via Mn^2+^ ions.

Figure 1B–D represent the 2D summary of interaction analysis results of CEN-BXA variants with I38T, I38F, and I38M mutations, respectively. BXA bound to each of the I38 mutation models engages with Tyr24 through pi-stacking interaction and Glu119 also hydrogen bonds with BXA in all mutation models. In the wild-type CEN protein (Fig. 1A), I38T (Fig. 1B) and I38M (Fig. 1D) mutation models, His41 interaction and interactions via Mn^2+^ ions are conserved. Similar to Ile38 (Fig. 1A), Met38 in the I38M mutation model (Fig. 1D) is placed adjacent to the aromatic ring of BXA during the MD simulation. However, Thr38 in the I38T model does not interact with the aromatic ring of BXA (Fig. 1B). This result indicates that the probability of interaction between BXA and Thr38 in the I38T mutation model is less than 30%. In Fig. 1C, Phe38 in the I38F mutation model shows different mode of interaction with BXA as compared to other mutation models. In the I38F mutation model, only one Mn^2+^ ion (Mn-A) is involved in the interaction with BXA, and the interaction between Mn-B and Glu80 is not conserved. Moreover, the number of interactions involving Asp108 and Glu119 via Mn^2+^ ions is reduced from 3 to 2 and the pi-stacking interaction with His41 is also abolished. Additionally, the mutated Phe38 residue forms a new pi-stacking interaction with BXA.

**Figure 2:**
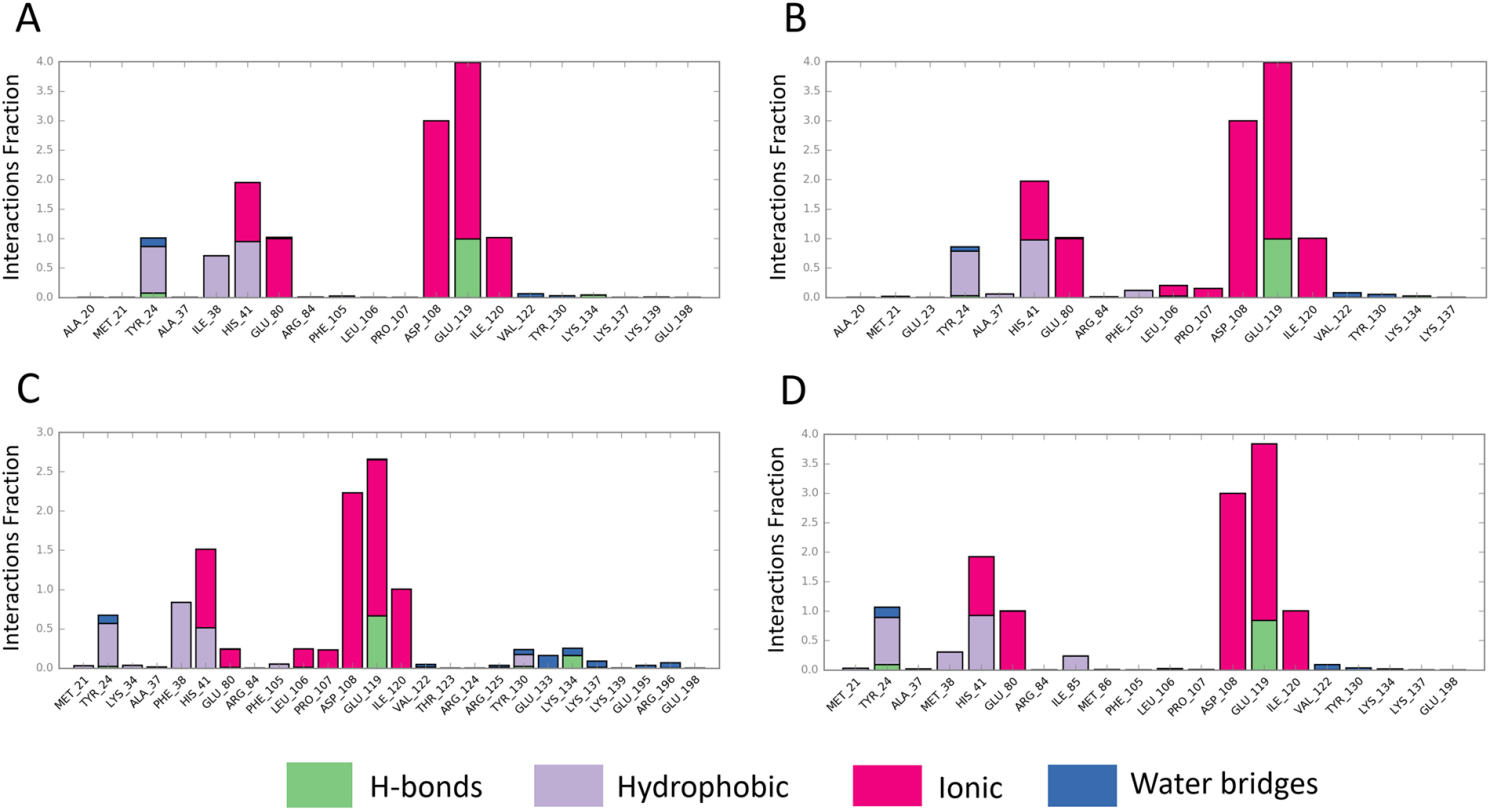
Interaction Fraction summary of CEN-BXA contacts. This graph is normalized by the total simulation time. Interaction-fraction values over 1.0 indicate that the residue has multiple contact routes to interact with the ligand. (**A**) Interaction fraction of BXA with the wild-type CEN protein (PDBID: 6FS6), (**B**) Interaction fraction of BXA with CEN having the I38T mutation (PDBID: 6FS7), (**C**) Interaction fraction of BXA with CEN having the I38F mutation, (**D**) Interaction fraction of BXA with CEN having the I38M mutation.

Figure 2 shows that the interaction fraction of CEN-BXA contacts during the entire simulation time. In Fig. 2A, a 0.708 interaction-fraction value of Ile38 suggests that a hydrophobic interaction was formed with a probability of 70.8% during the simulation. Compared to the wild-type model, the I38T model also shows similar interaction-fraction value (Fig. 2B) with the exception of the hydrophobic interaction as seen in the wild-type model (Fig. 2A). Similar to the wild-type model, the interaction pattern is conserved in the I38M mutation model (Fig. 2D). Although, Met38 forms hydrophobic interaction with BXA, the interaction-fraction value of Met38 is 0.305 as compared to 0.708. In contrast, Phe38 in the I38F model maintains hydrophobic interactions during the simulation, although the number of polar interactions via the Mn^2+^ ions is reduced. Phe38 in the I38F model presents an interaction-fraction value of 0.837, which is higher than that of the wild-type model. Moreover, the ionic interaction-fraction values of Asp108 (2.232) and Glu-119 (1.988) in the I38F model are less than 3.0.

**Figure 3:**
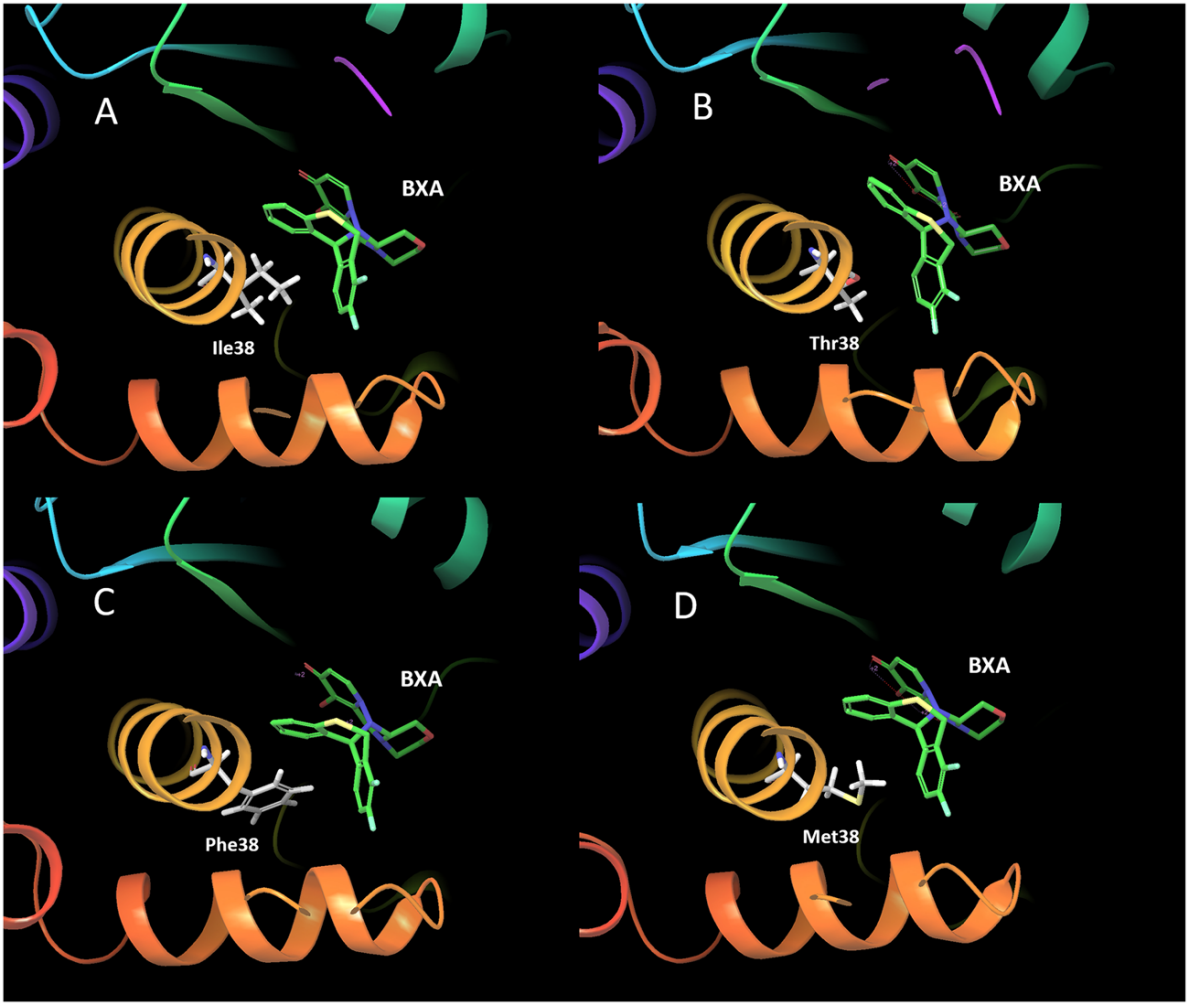
BXA binding site of representative structures with highest population in MD simulation. (**A**) Binding site of BXA depicting interaction between the ligand and Ile38 residue in CEN (PDB entry: 6FS6, Population ratio: 0.163), (**B**) Binding site of BXA depicting interaction between the ligand and Thr38 residue in CEN (PDB entry: 6FS7, Population ratio: 0.434), (**C**) Binding site of BXA depicting interaction between the ligand and Phe38 residue in CEN (Population ratio: 0.263), (**D**) Binding site of BXA depicting interaction between the ligand and Met38 residue in CEN (Population ratio: 0.323).

To confirm the CEN-BXA complex with the highest population in each MD simulation, we conducted a trajectory clustering analysis. Figure 3 shows the representative structure of the CEN-BXA complex with the highest population obtained through MD simulation. Figure 3A shows the BXA binding site of the representative structure depicting the interaction between BXA and Ile38 of CEN. In this figure, the alkyl chain of Ile38 is adjacent to the aromatic ring of BXA. This indicates that the alkyl side chain of Ile38 interact with the aromatic ring of BXA via CH-pi interaction, which is classified as hydrophobic interaction. Figure 3B–D show the spatial location of Thr38, Phe38 and Met38 residues in the BXA binding site in the I38 mutation model representative structures. Figure 3B shows that both Ile38 and Thr38 are equidistant from the aromatic ring of BXA, while the phenyl group of Phe38 and the methyl sulfide group of Met38 are adjacent to the aromatic ring of BXA. These data indicate that the phenyl group of Phe38 and the methyl sulfide group of Met38 interact via T-stacking and CH-pi interaction, respectively, with the aromatic ring of BXA.

### Binding free energy analysis by MM/GBSA

To compare how I38 mutations in CEN affect the binding free energy of BXA, we used the MM/GBSA method using the wild-type (I38) and all the mutation models. Interaction analysis information of the mutation models are presented in Supplementary Figs. 2 and 3. Table 1 lists the ΔG_bind_ of BXA for the wild-type and all the mutation models. The binding free energy of BXA for the wild-type CEN protein with I38 is −8.97 kcal/mol. Compared to I38, binding free energies of BXA for I38T and I38M variants, which show decreased affinity towards BXA are energetically less favorable (ΔG_bind_ of −2.30 and −0.65 respectively). However, the binding free energy of I38F variant, which also shows a reduced affinity for BXA is more favorable (ΔG_bind_ −14.84 kcal/mol). Moreover, we also predicted the variation in the binding free energy for the other 16 mutation models. The binding free energies of BXA in the I38N, I38C, I38P and I38W mutation models are energetically more favorable as compared to that of BXA in the wild type; other mutations models show less favorable binding energies. Among all models, the I38P mutation model demonstrated the most favorable and the I38R mutation model, the least favorable binding free energy value.

**Table 1:** Binding free energy of BXA for the wild-type CEN and all the I38 mutation models.

Mutation	ΔG_bind_	Mutation	ΔG_bind_
WT	−8.97	I38Q	0.59
I38T	−2.3	I38E	0.96
I38F	−14.84	I38H	9.08
I38M	−0.65	I38L	−6.9
I38A	−6.52	I38K	2.17
I38G	−7.6	I38P	−14.91
I38R	9.45	I38W	−11.33
I38N	−9.01	I38V	−5.37
I38D	−5.71	I38Y	0.29
I38C	−8.99	I38S	−4.51

## Discussion

**Figure 4:**
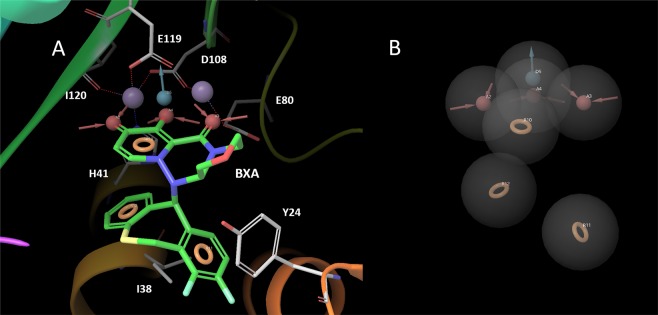
Pharmacophore feature of BXA for binding to CEN as estimated by MD simulation. Red feature: Hydrogen bond acceptor (lone pair), Blue feature: Hydrogen bond donor, Orange feature: Aromatic ring, (**A**) The BXA binding site in CEN with important residues represented as sticks, BXA and pharmacophore feature. (**B**) The pharmacophore feature of BXA is displayed.

In this study, we performed MD simulations on CEN-BXA complexes with 20 variants of 38th amino acid residue to investigate the sensitivity of BXA by mutation of I38. MD simulation results suggest that BXA interacts with Tyr24 and His41 of CEN via pi-stacking, and also forms hydrogen bonds with Glu119. Ile38 is placed adjacent to the aromatic ring of BXA during the MD simulation. Furthermore, BXA interacts with Glu80, Asp108, and Ile120 through two Mn^2+^ ions. Asp108 and Glu119 have 3 ionic interaction routes that connect them with BXA, while His41, Glu80, and Ile120 have 1 ionic interaction route connecting them to BXA. Figure 4A,B show the pharmacophore model for BXA binding to CEN. Lone pair electrons of the carbonyl and hydroxyl group of BXA form ionic interactions with His41, Glu80, Asp108, Glu119 and Ile120 via Mn^2+^ ions. Therefore, the lone pair of electrons that have the property of being a hydrogen bond acceptor is important for binding to CEN, also, three characteristic interactions involving the lone pair of electrons are required for inhibiting CEN (Fig. 4). Aromatic ring structures with fluorine atoms or hydroxyl group are important for pi-stacking and CH-pi interactions with Tyr24, Ile38 and His41, and the hydroxyl group of BXA hydrogen bonds with Glu119. Thus, the aromatic pharmacophores and hydrogen bond donor feature are also required for binding of BXA to CEN. Consequently, interaction analysis by MD simulation suggested that these characteristics of BXA must be conserved while optimizing inhibitors against the drug resistant variants.

The MD simulation results for the mutation models show that the I38T mutation results in a loss of interaction between Thr38 and BXA. Moreover, the interaction-fraction value of Met38 is lower than that of I38. The side chain methyl group of threonine is connected to C_α_ atom via two single bonds. In the case of methionine, 4 single bond interactions connect the methyl group with C_α_ atom. In contrast, the side chain methyl group of isoleucine is connected to C_α_ atom via three single bond interactions. Therefore, the methyl group of Thr38 is at a distance too far to interact with BXA, and methyl group of Met38 is too close to interact with BXA. In the I38F mutation model, MD simulation suggests that although Phe38 interacts with aromatic ring of BXA by T-stacking interaction, one of the ionic interaction bridges via Mn^2+^ ion is abolished. As a result, I38T, I38F and I38M mutations, which have been reported to exhibit reduced BXA sensitivity, have a lesser number and probability of interactions as compared to those of the wild-type protein. MD simulation results suggest that BXA is highly sensitive to the mutation of I38 because the methyl group with proper distance is essential to form the CH-pi interaction between CEN and BXA.

To predict variation in the binding free energy owing to I38 mutation, we performed MM/GBSA simulation using the WT CEN-BXA complex model and all the I38 mutation models. As a result, except for I38F, all the other I38 mutation models, which exhibit reduced BXA sensitivity, show less favorable binding free energy as compared to the wild type CEN. I38R demonstrated the least favorable binding free energy value. Arginine has a long flexible side chain as compared to isoleucine. The binding free energy of I38K, with physical properties similar to I38R was also energetically less favorable as compared to the wild-type (I38) protein. On the other hand, amino acid residues such as alanine and valine, which have a methyl group necessary for CH-pi interaction also showed less favorable binding free energy as compared to I38. Although I38F mutations have been reported to exhibit reduced BXM sensitivity, binding free energy of I38F estimated by MM/GBSA simulation is lower than that of WT. In this paper, the MMGBSA method is used to predict the binding free energy between BMX and each CEN model, and the thermodynamic stability between the models are compared. However, MM/GBSA simulation cannot consider to kinetic stability such as energy barrier which occurs when BXM binds to CEN. Therefore, these results conceivable that I38F mutation may reduce the affinity of BXM for CEN by destabilizing energy barrier. Interaction analysis shows that a proper distance needs to be maintained for the CH-pi interaction to occur between the aromatic ring of BXA and the 38^th^ amino acid residue. The methyl group of Ile38 is placed at a distance optimal for the CH-pi interaction to happen. MD simulation and MM/GBSA results suggest that I38 mutations that result in the elimination of hydrophobic interactions with the aromatic ring of BXA, exhibit reduced BXA sensitivity. Therefore, 14 mutations out of 19 possible mutations are potential drug resistant mutations.

The interaction analysis results obtained from MD simulation and MM/GBSA for CEN-BXA complex models provide information regarding protein-ligand interactions and possible mechanism of drug resistance of influenza virus. These findings of binding mechanism would be crucial for future drug optimization and may prove useful for development of new antiviral drugs against this infectious agent.

## Methods

### Preparation for CEN-BXA complex model

The wild-type and I38 mutant CEN-BXA complex structures (PDB entries: 6FS6 and 6FS7) were obtained from the Protein Data Bank. Except for I38T variant, all the other mutation models were created using “Sequence viewer” in Maestro^37^ and were based on 6FS6 structure. Assignment of bond orders and hydrogenation for the CEN-BXA complex structure were performed using Protein Preparation in Maestro. The ionization state of the BXA suitable for pH 7.0 ± 2.0 was predicted using Epik^38^. H-bond optimization was conducted using PROPKA^39^. Energy minimization was performed using the OPLS3e force field^40^.

### MD simulation

MD simulations for interaction analysis and evaluation of binding free energy were performed using Desmond^41^. All systems were set up using “System Builder” in Maestro. The wild-type CEN-BXA complex structure and all the I38 mutation models, which were subjected to energy minimization, were placed in the orthorhombic box with a buffer distance of 10 Å in order to create a hydration model. SCP water model^42^ was used for creation of the hydration model. The cut-off radius for van der Waals, time step, initial temperature and pressure of the system were set to 9 Å, 2.0 fs, 300 K and 1.01325 bar respectively. Desmond evaluates electrostatic force by dividing near term and far term, and the boundary between near term and far term is 9 Å^43^. Moreover, the sampling interval during the simulation was set to 100 ps. Finally, we performed MD simulations under the NPT ensemble for 1 μs. The initial structure of each MD simulation can be downloaded from the following link: https://github.com/sekijima-lab/MMGBSA-CEN-BXM.

### Interaction analysis and trajectory clustering for MM/GBSA

 To analyze these MD simulations, the Simulation Interactions Diagram tool in Maestro was used to perform an interaction analysis between CEN and BXA, and the Desmond trajectory clustering tool was used to obtain representative structures for evaluating binding free energy. In trajectory clustering, backbone-atom was set for root mean square deviation (RMSD) matrix and the analysis was performed through the affinity propagation clustering method^44^. “Trajectory Frame Clustering” in Maestro was used to estimate the most populated representative structure for each MD simulation. Trajectory frame extraction interval was 10 frames, and 1000 frames were used for clustering each trajectory, and maximum output number of clusters was set to 10. The initial structure of each MD simulation can be downloaded from the following link: https://github.com/sekijima-lab/MMGBSA-CEN-BXM. The structure with the largest number of neighbors in the structural cluster was used for binding free energy calculation as the representative structure. Calculation of the binding free energy was conducted by Prime MMGBSA tool in Maestro. The VSGB solvation model^45^ and OPLS3e force field were set for binding free energy calculation. Representative structures with highest population were used.

## Supplementary information

Supplementary Information
