# Supplementary material for: Molecular Dynamics Simulation reveals the mechanism by which the Influenza Cap-dependent Endonuclease acquires resistance against Baloxavir marboxil
Source: Sci Rep. 2019 Nov 25;9:17464. doi: 10.1038/s41598-019-53945-1 (PMC6877583; doi:10.1038/s41598-019-53945-1)
Supplement: Supplementary file 1 — Supplementary Information [file 41598_2019_53945_MOESM1_ESM.docx]

### Title

Molecular dynamics simulation reveals the mechanism by which the Influenza Cap-dependant Endonuclease acquires resistance against Baloxavir marboxil

**Authors**

Ryunosuke Yoshino^1,2^, Nobuaki Yasuo^3^, Masakazu Sekijima^3*^

**Affiliations**

^1^Transborder Medical Research Center, University of Tsukuba, 1-1-1 Tennodai, Tsukuba, Ibaraki 305-8577, Japan

^2^Center for Computational Sciences, University of Tsukuba, 1-1-1 Tennodai, Tsukuba, Ibaraki 305-8577, Japan

^3^Advanced Computational Drug Discovery Unit, Tokyo Institute of Technology, J3-23-4259 Nagatsutacho, Midori-ku, Yokohama 226-8501, Japan^3^

*Corresponding author


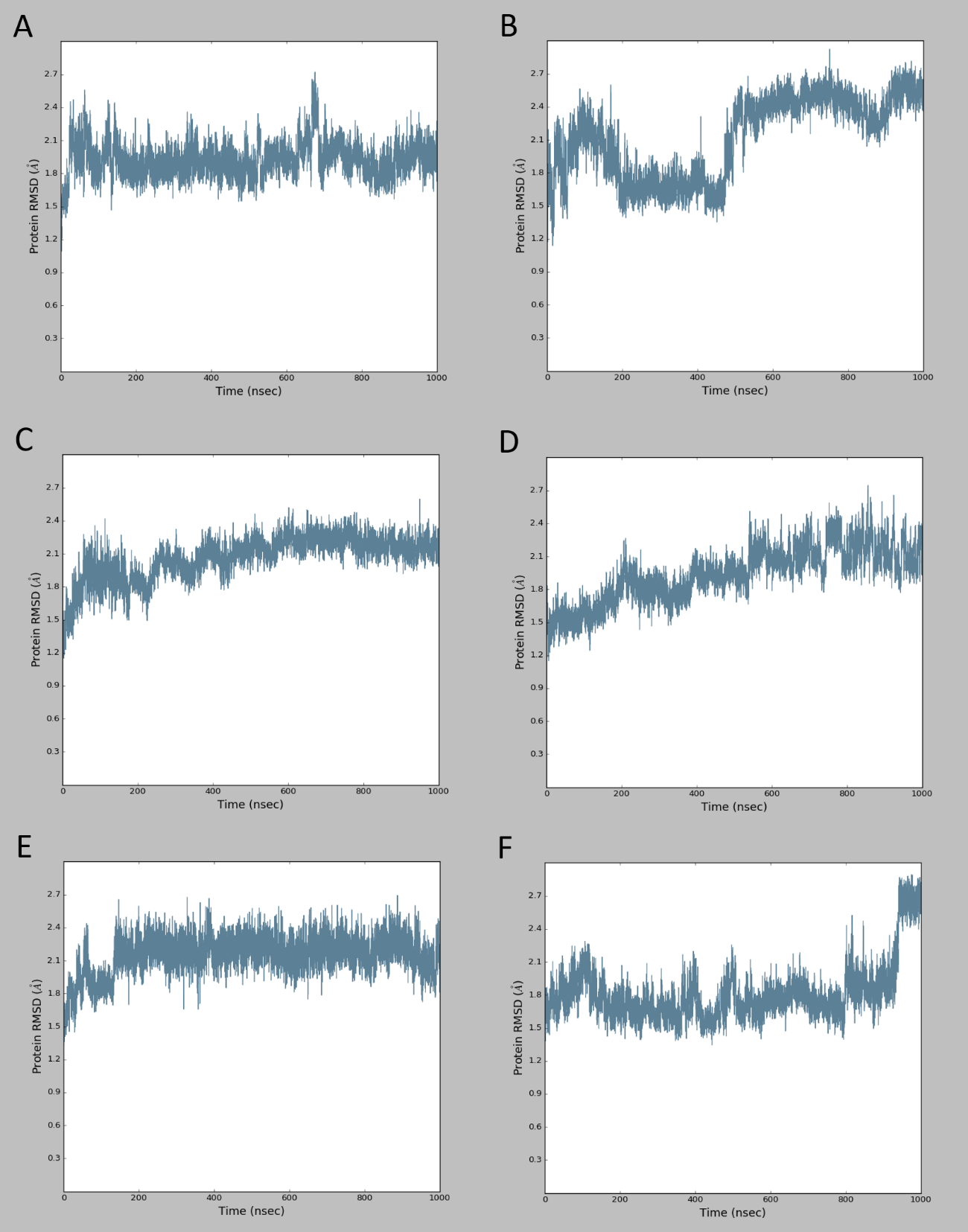


Figure S1. Root mean square deviations of CEN C_α_ atoms in the MD simulations. A: WT (I38), B: I38T, C: I38F, D: I34M, E: I38A, F: I38G


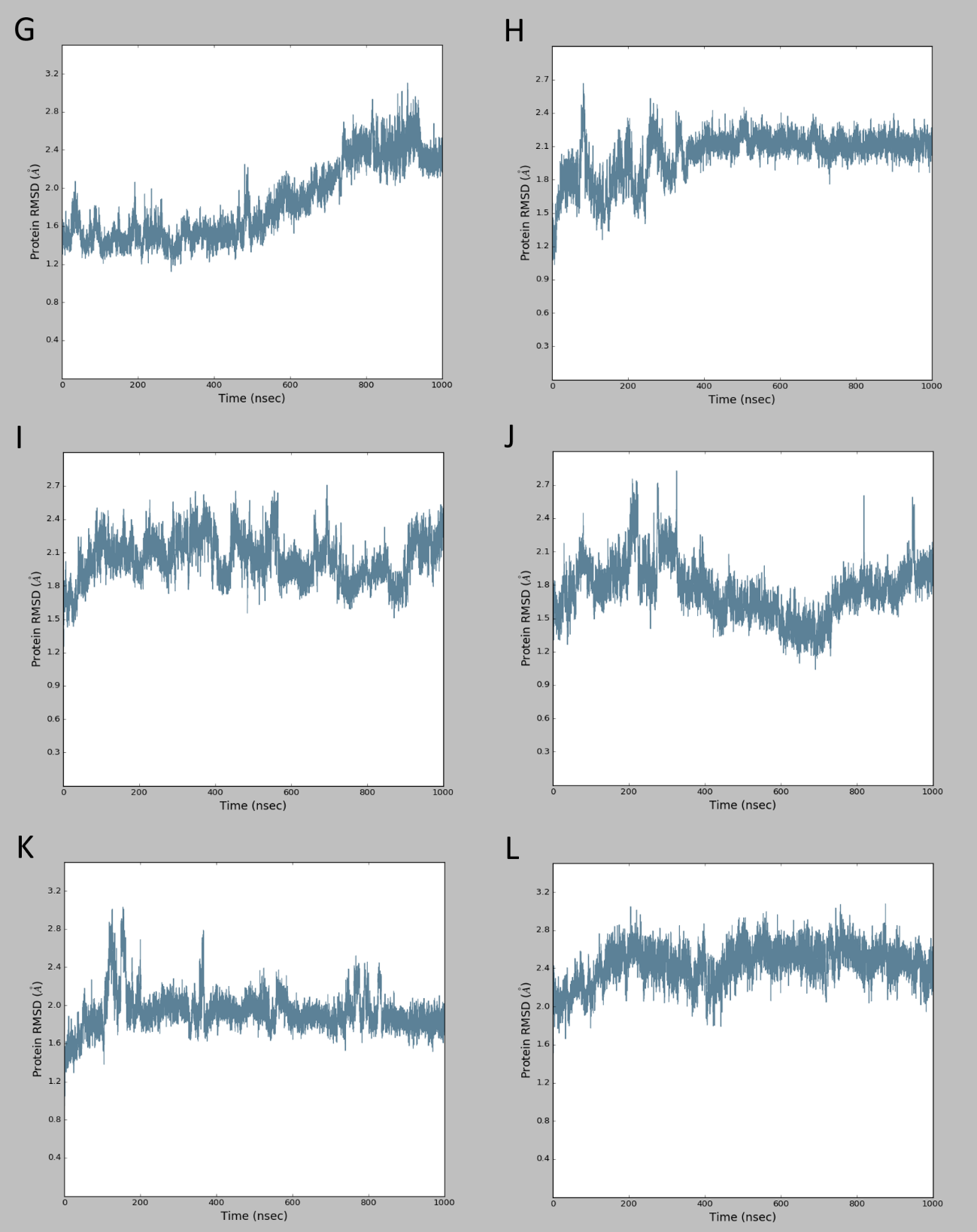


Figure S1. Continued. G: I38R, H: I38N, I: I38D, J: I38C, K: I38Q, L: I38E


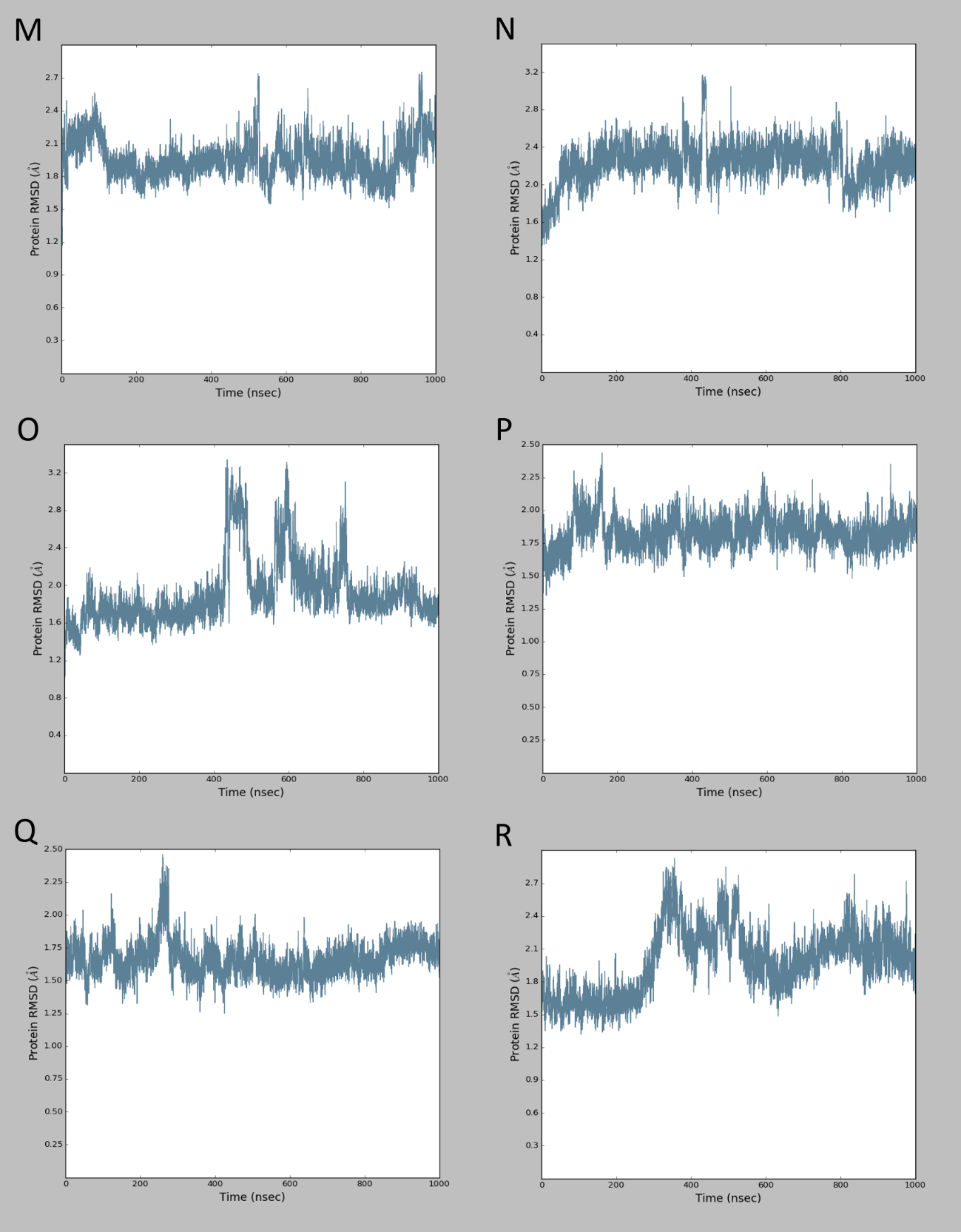


Figure S1. Continued. M: I38H, N: I38L, O: I38K, P: I38P, Q: I38W, R: I38V


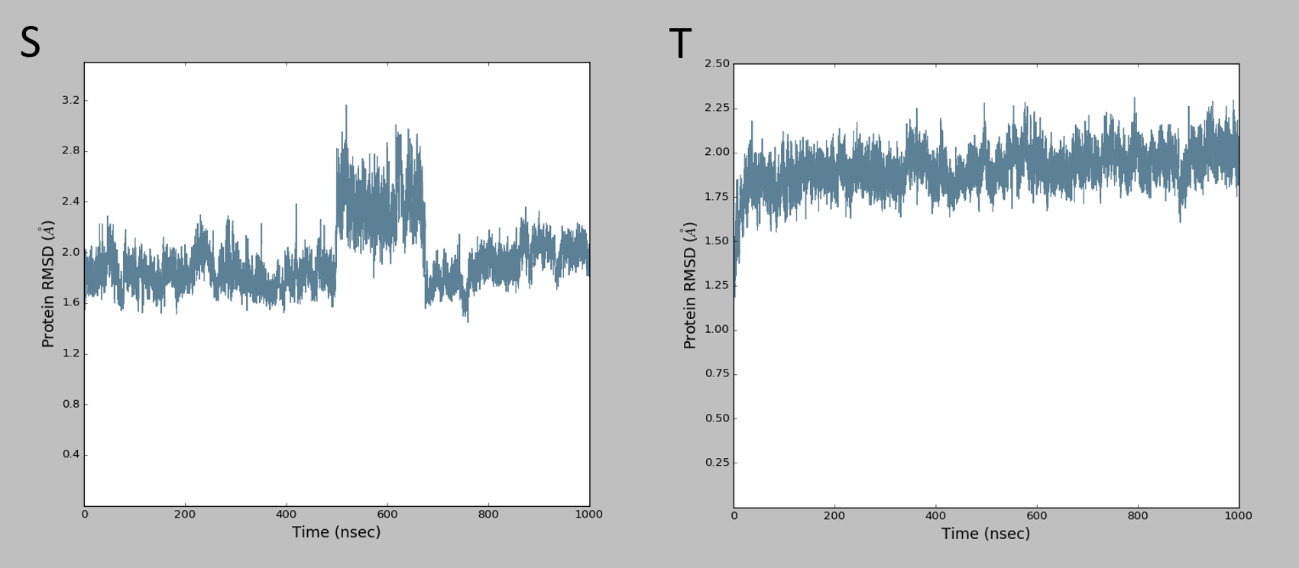


Figure S1. Continued. S: I38Y, T: I38S


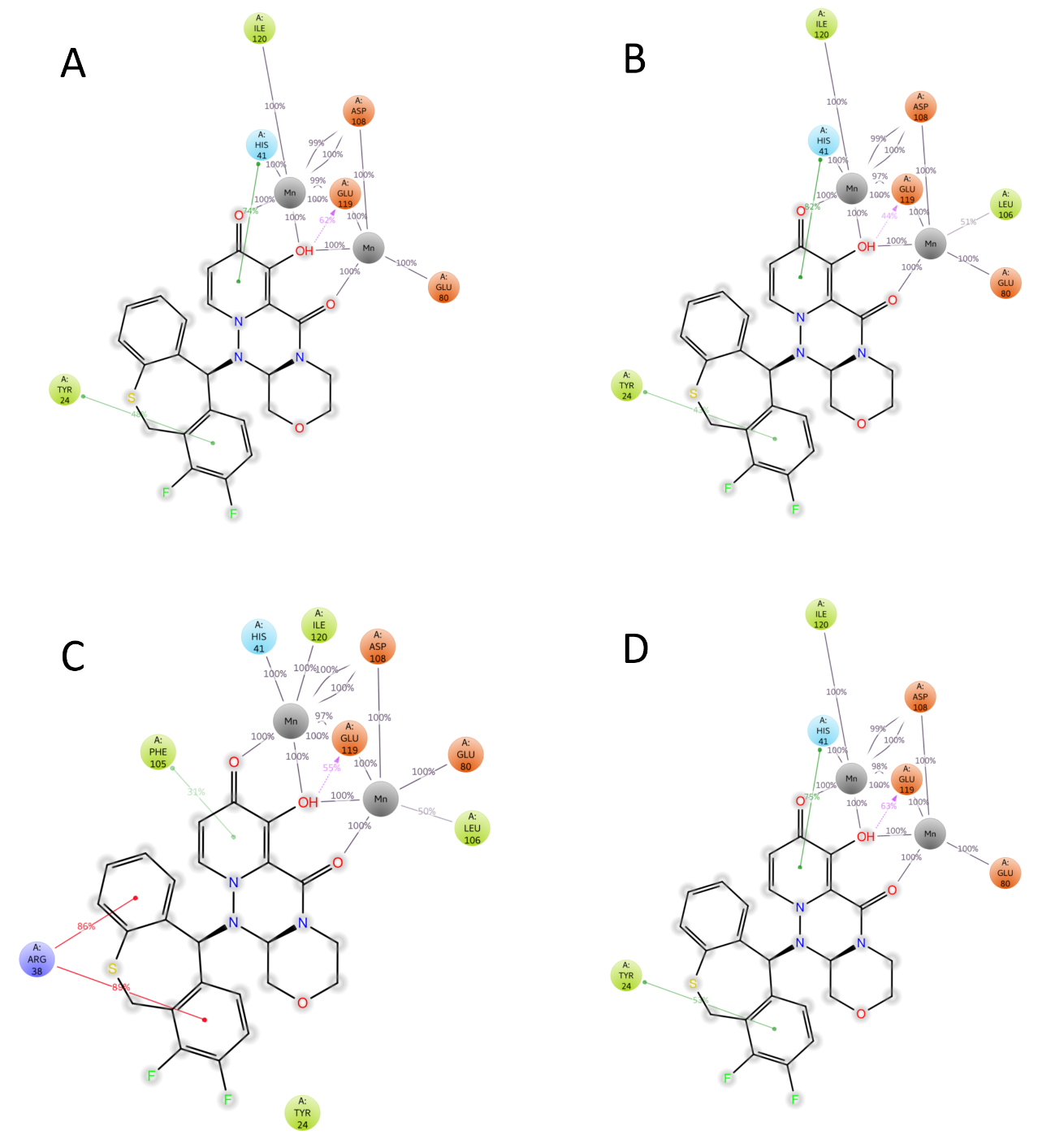


Figure S2. 2D summary of interaction analysis results of CEN-BXA. The interaction pairs that occur during more than 30% of the simulation time are included. A: I38A, B: I38G, C: I38R, D: I38N mutation models


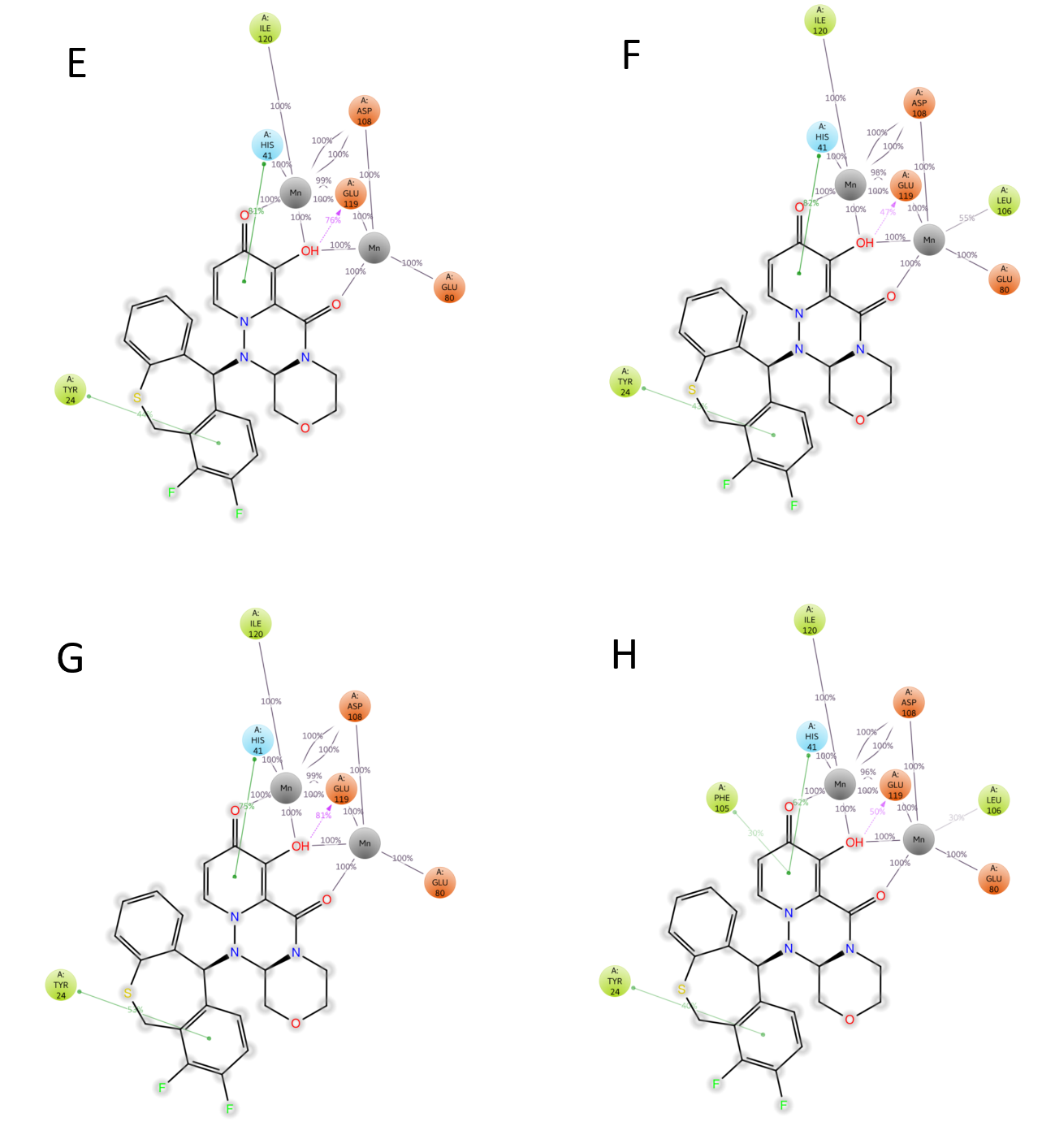


Figure S2. Continued. E: I38D, F: I38C, G: I38Q, H: I38E


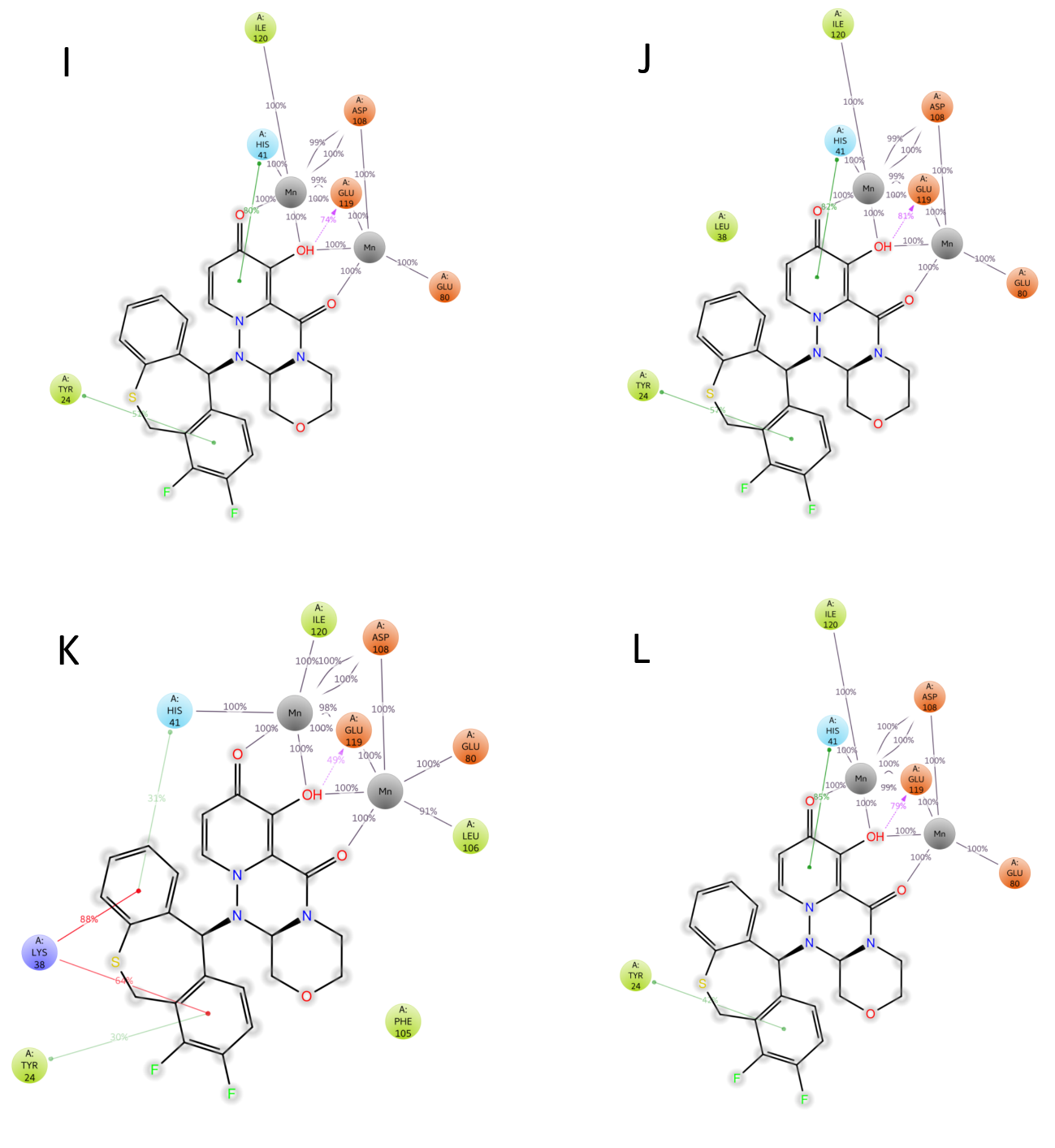


Figure S2. Continued. I: I38H, J: I38L, K: I38K, L: I38P


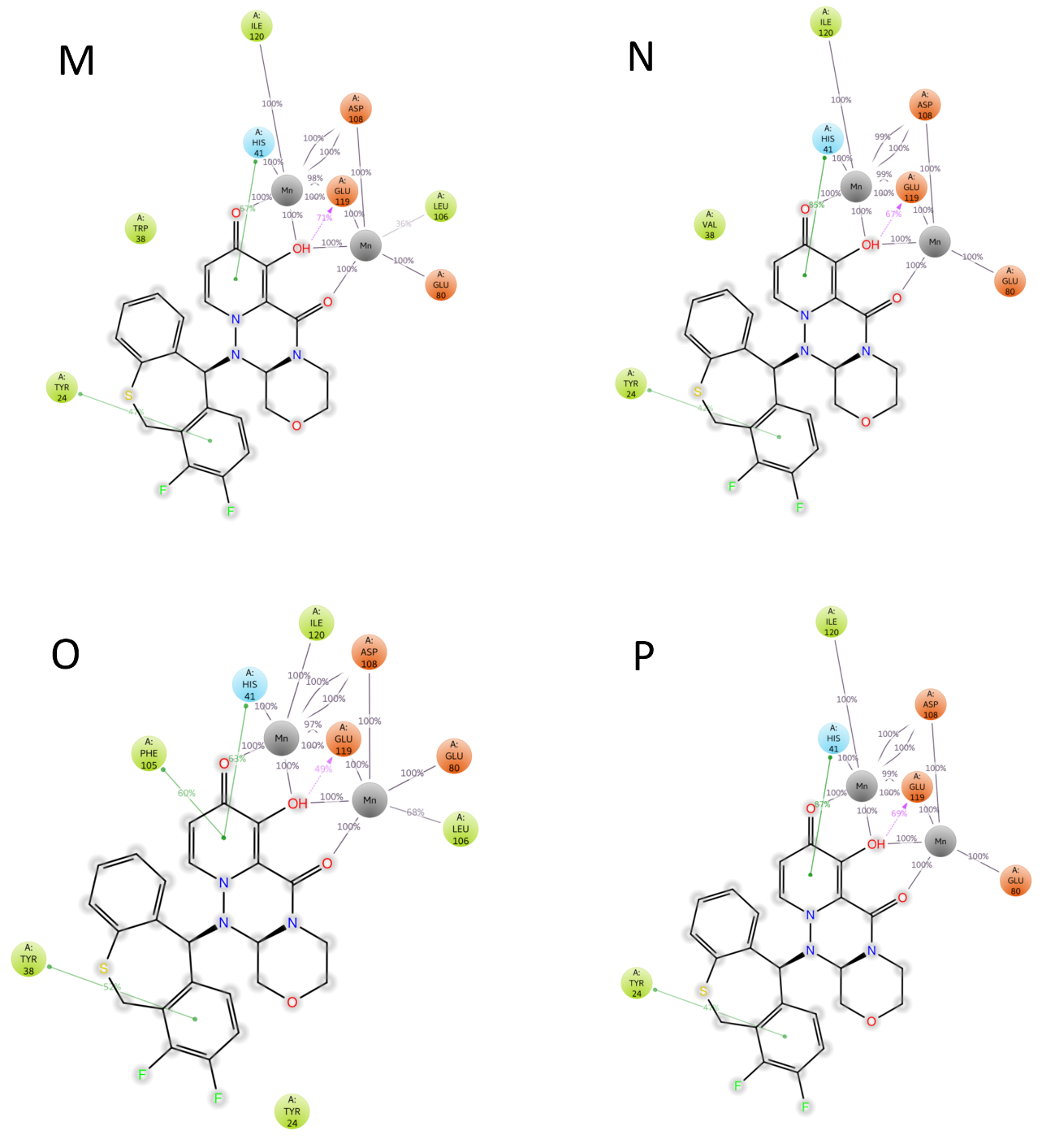


Figure S2. Continued. M: I38W, N: I38V, O: I38Y, P: I38S


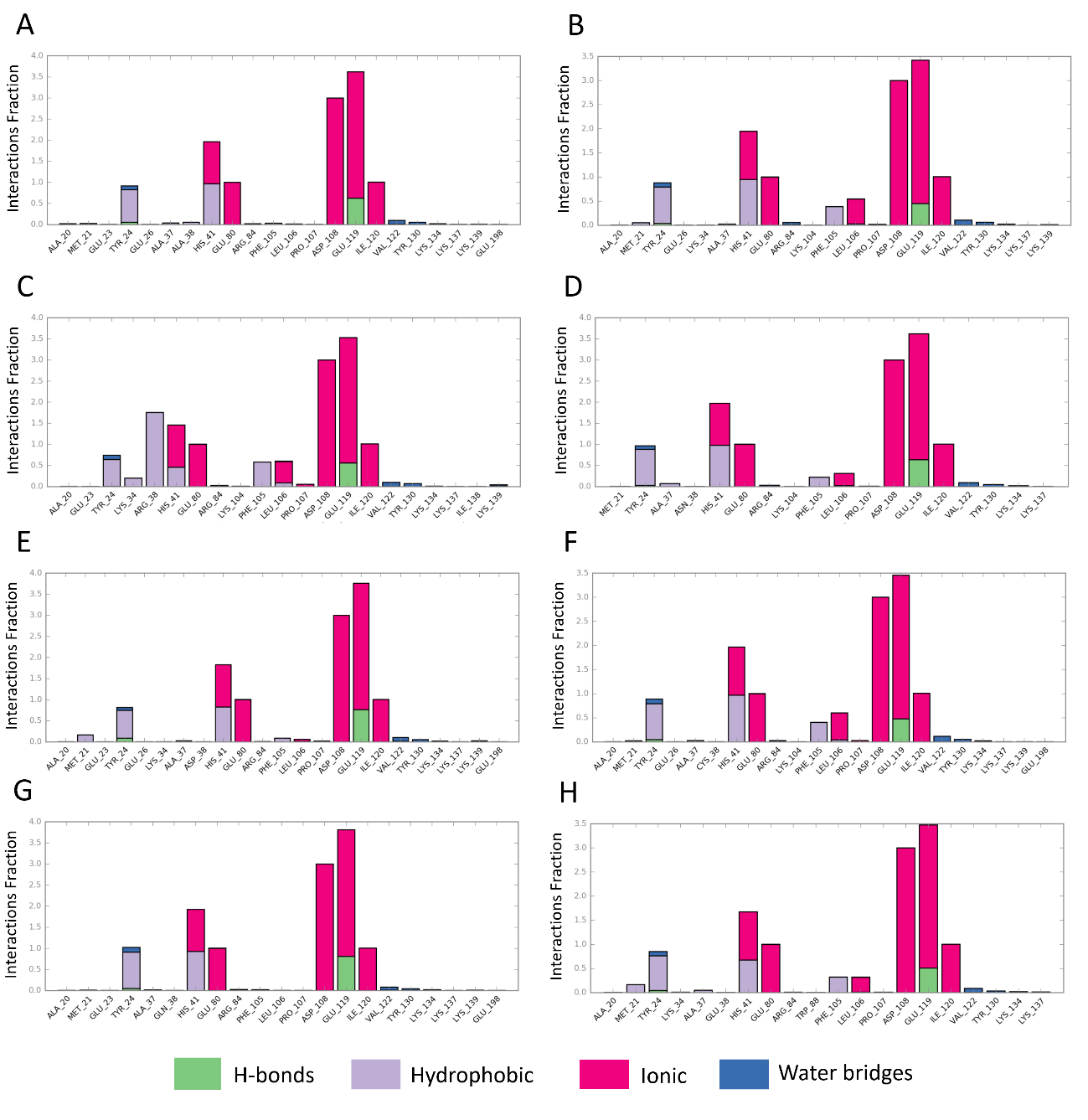


Figure S3. Fraction summary of CEN-BXA contacts. These graphs have been normalized with respect to the total simulation time. Interaction-fraction values over 1.0 indicate that the residue interacts with the ligand through multiple contact routes. A: I38A, B: I38G, C: I38R, D: I38N, E: I38D, F: I38C, G: I38Q, H: I38E


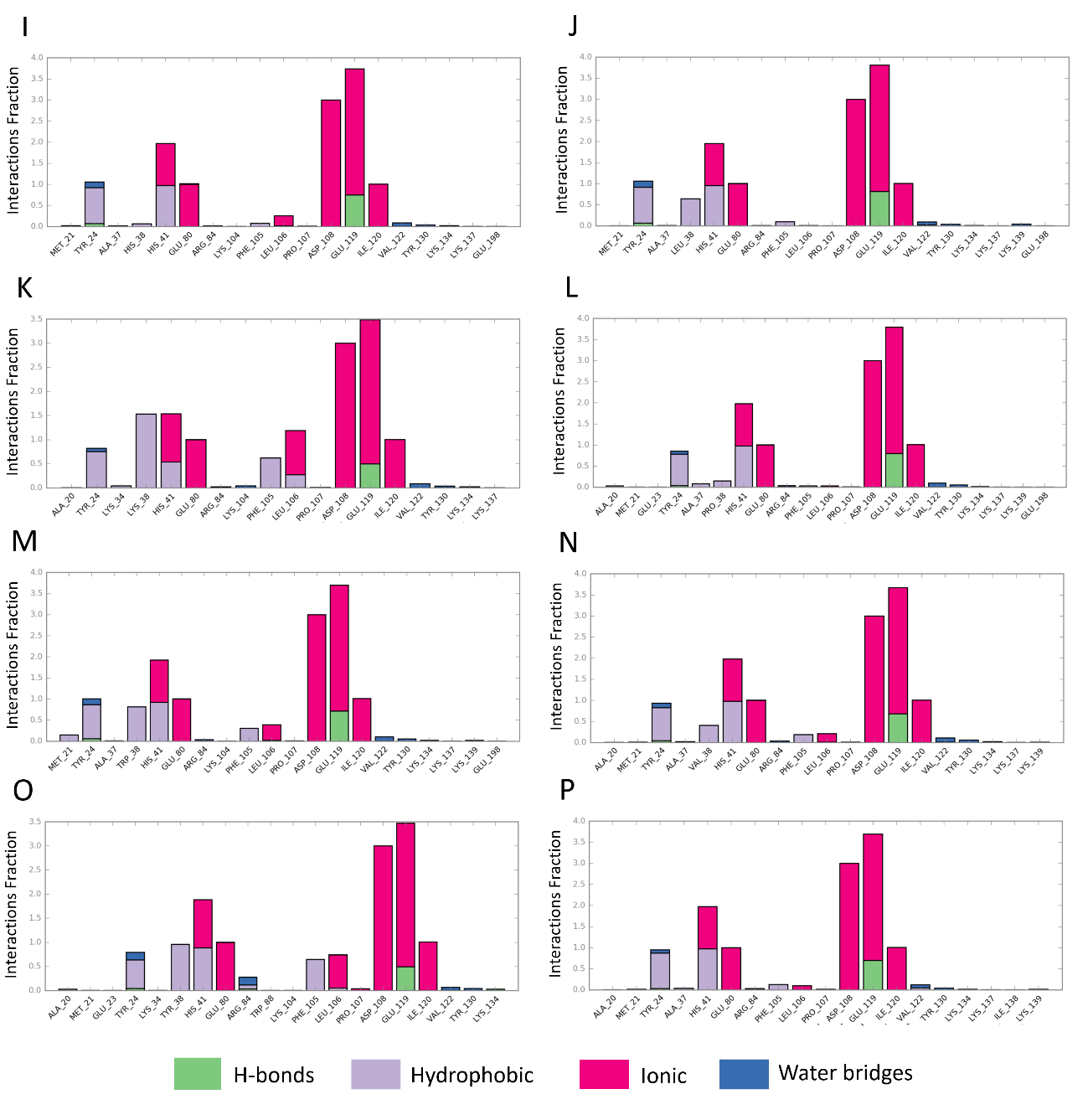


Figure S3. Continued. I: I38H, J: I38L, K: I38K, L: I38P, M: I38W, N: I38V, O: I38Y, P: I38S
